# Association between time to surgery and survival in patients with initially diagnosed WHO 2021 grade 4 gliomas

**DOI:** 10.1093/noajnl/vdag133

**Published:** 2026-05-20

**Authors:** Haruka Kanamitsu, Eiichi Ishikawa, Shunichiro Miki, Narushi Sugii, Masahide Matsuda

**Affiliations:** Department of Neurosurgery, Institute of Medicine, University of Tsukuba, Ibaraki, Japan; Department of Neurosurgery, Institute of Medicine, University of Tsukuba, Ibaraki, Japan; Department of Neurosurgery, Institute of Medicine, University of Tsukuba, Ibaraki, Japan; Department of Neurosurgery, Institute of Medicine, University of Tsukuba, Ibaraki, Japan; Department of Neurosurgery, Institute of Medicine, University of Tsukuba, Ibaraki, Japan

**Keywords:** doubling time, high-grade glioma, maximal safe resection, time to surgery

## Abstract

**Background:**

Glioblastoma (GBM) and other WHO 2021 grade 4 gliomas are highly aggressive tumors treated with maximal safe resection followed by chemoradiation. Although these tumors grow rapidly, the optimal timing of surgery remains unclear. This study evaluated whether time to surgery (TTS), defined as the interval from first admission to surgery, affects prognosis in patients with grade 4 glioma.

**Methods:**

A total of 219 patients with grade 4 glioma were divided into 4 TTS groups (≤ 3 days, 4-7 days, 8-14 days, ≥ 15 days). Clinical data, including age, sex, tumor location, preoperative and postoperative Karnofsky Performance Status (KPS), KPS at discharge, time from hospitalization to surgery, and extent of resection (EOR), and other various factors, were collected from electronic medical records.

**Results:**

Patients who underwent surgery within 3 days had significantly lower preoperative KPS, whereas longer TTS was associated with higher KPS. Overall survival slightly longer in the group with longer TTS. However, among patients who achieved EOR ≥ 90%, TTS showed no association with survival. Multivariate analysis identified the following independent predictors of better prognosis: higher postoperative KPS, non-GBM histology, achievement of EOR ≥ 90%. TTS itself was not a prognostic factor.

**Conclusions:**

While urgent surgery is necessary for patients with rapid neurological deterioration, it is important to plan surgery at an appropriate time that allows for preservation of KPS and achievement of maximal safe resection.

Key Points

- Overall survival was slightly longer in GBM patients with longer TTS.
- In cases of EOR ≥ 95%, TTS showed no association with survival.
- Multivariate analysis showed TTS was not an independent prognostic factor.

Importance of the Study

This study clarified the clinical significance of time to surgery (TTS) in patients with WHO grade 4 glioma, since previous ­literature in this field had shown inconsistent results. Analysis of 219 cases with detailed clinical data revealed that, considering preoperative status and resection margin, TTS is not an independent prognostic factor. These findings suggest that, except in cases with severe intracranial pressure elevation, optimizing the patient’s condition and planning for safe maximal resection may contribute to improved survival rates rather than rushing surgery. This study provides evidence to guide timing decisions and supports future research on individualized perioperative strategies that balance urgency with functional preservation.

Glioblastoma (GBM) and other grade 4 gliomas, as defined by the World Health Organization (WHO) 2021 classification, are primary malignant brain tumors known for their poor prognosis. The standard treatment for GBM is maximal tumor resection followed by radiation therapy with temozolomide,^1^ and the same treatment is considered standard therapy for other grade 4 gliomas as well. The extent of resection (EOR) is crucially important for patient outcomes.^2^ However, surgeons must consider preventing neurological deficits after surgery, such as hemiparesis, aphasia, and apraxia, because GBM invades the functional cortex (eloquent areas) and the white matter containing critical fiber tracts. Therefore, when planning surgical procedures, neurosurgeons must balance maximal resection with the preservation of brain function. Recent advances in imaging and auxiliary diagnostic systems, used before or during surgery, have enabled more aggressive resections.^3^

Neurosurgeons tend to plan for the earliest possible removal due to the rapid progression of the disease, but there is no clear consensus on the optimal timing for surgery. Previous studies have shown that urgent surgery for high-grade glioma or GBM is associated with poor prognosis.^4^^,^^5^ On the other hand, Müller et al demonstrated that the time between the first diagnostic magnetic resonance imaging (MRI) and tumor resection (time to surgery, TTS) was not related to overall survival (OS).^6^ They also found that lower Karnofsky Performance Status (KPS) and larger tumor volume were associated with shorter TTS.^6^ In this study, we examined how TTS influenced prognosis in patients with WHO 2021 grade 4 gliomas (mainly GBM) at our hospital and discussed the optimal timing for tumor resection.

## Methods

### Patient Selection

A total of 219 patients who were initially diagnosed with GBM or other WHO 2021 grade 4 gliomas and underwent surgery at the University of Tsukuba Hospital between January 2018 and March 2024 were included in this retrospective study. Patient information, including age, sex, tumor location, preoperative (at first admission) and postoperative KPS, KPS at discharge, TTS, and EOR, was collected from the electronic medical records. This research protocol was approved by the Ethics Committee of the University of Tsukuba Hospital (Approval Number: R01-202). Registered patients were provided with an opt-out consent form posted on our institution’s website to protect individual privacy. The requirement for obtaining written informed consent was waived by the aforementioned Ethics Committee. Regarding EOR, we defined “EOR 100%” as complete resection of the contrast-enhancing lesion (100%), “EOR 90-99%” as minimal residual contrast enhancement (≥ 90% resection or residual lesion ≤ 10 mm in maximum diameter), biopsy as specimen collection without tumor volume reduction, and partial resection (PR) as cases falling between “EOR 90-99%” and biopsy. In this study, TTS was defined as the period from the first hospital admission to surgery and was classified into four groups: 3 days or less (≤ 3 days), 4-7 days, 8-14 days, and 15 days or more (≥ 15 days). Almost all glioma patients were initially admitted within one week of their initial outpatient visit, and most patients underwent surgery during this first hospitalization; however, a small number were discharged and subsequently underwent surgery during a second hospitalization. The first diagnostic MRI was usually performed on the day of the first admission or the following day.

### Tumor Measurements and Growth Rate Calculations

We examined the growth rate of contrast-enhancing lesions, including areas of central necrosis, in 41 cases in which repeated MRI scan was performed at least 10 days after the initial head MRI and prior to surgical resection. Tumor volume was calculated using contrast-enhanced T1-weighted images from the initial MRI and the MRI performed immediately prior to surgery, employing the 3D image analysis system SYNAPSE VINCENT v7.0.0003 (Fujifilm, Co., Japan). Cases with a primary lesion that did not enhance or showed minimal enhancement were excluded from the analysis.

Additionally, in cases in which tumor volume could be calculated, tumor doubling time was computed as follows. Here, “dt” represents the number of days between MRI examinations, “V1” represents the volume at the initial head MRI examination, and “V2” represents the tumor volume at the MRI examination performed immediately before surgery^7^:


VDt＝(dt*ln(2))/ln(V2⁄V1) (day)

### Statistical Analysis

All statistical analyses were performed using SPSS Statistics version 28.0.0.0 (IBM Corporation, Armonk, New York, USA). Chi-square tests were used for categorical variables, such as tumor location and EOR, while the Kruskal-Wallis test was used to compare KPS scores between groups. Simple linear regression analysis was performed to examine the relationship between the number of days from first admission to surgery and KPS. Differences in EOR between groups were analyzed using the Mann-Whitney U test. OS was evaluated for each group using Kaplan-Meier curves, and differences were assessed with the log-rank test. Factors affecting survival prognosis were evaluated using Cox proportional hazards models.

## Results

### Patient Characteristics

**Figure 1.**
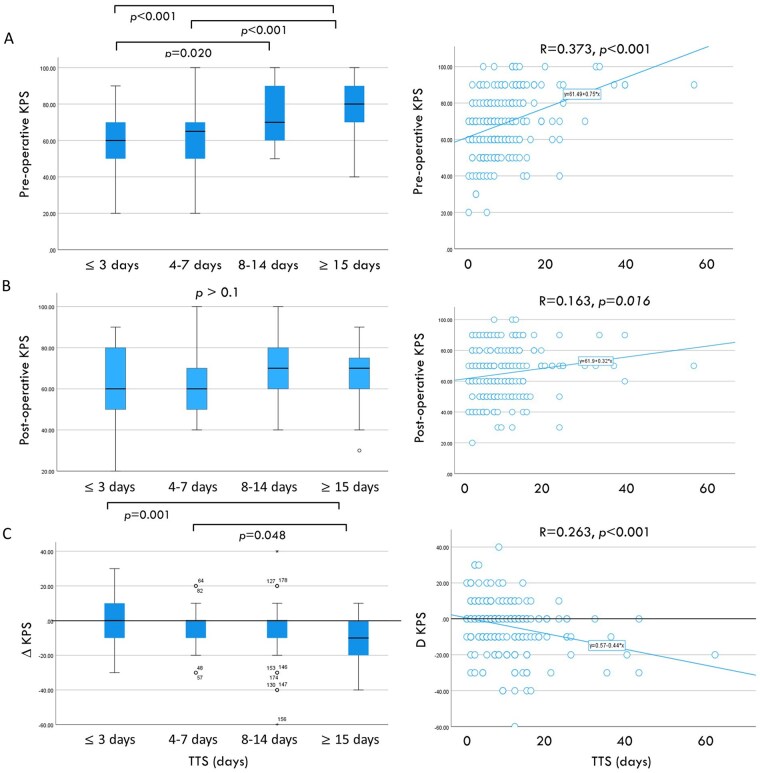
Association between time to surgery (TTS) and Karnofsky Performance Status (KPS). (A) Preoperative KPS, (B) Postoperative KPS, (C) ΔKPS (postoperative KPS − preoperative KPS). The Kruskal-Wallis test with Bonferroni correction and simple linear regression analysis were performed for the left and right panels, respectively.

Table 1 summarizes the baseline characteristics of the cases. The median age was 69 years, and the median time from admission to surgery was 8 days (average; 9.6 days). The number of patients in the ≤ 3-day, 4-7-day, 8-14-day, and ≥ 15-day groups were 44, 62, 74, and 39, respectively. Of the 219 cases, 180 (82%) and 212 (97%) patients underwent craniotomy within 14 days and within 1 month of first admission, respectively. Analysis of tumor types between the ≥ 15-day and the less-than-15-day (< 15-day) group revealed that typical GBM, specifically IDH wildtype GBM, was less common in the ≥ 15-day cases (Supplementary Figure S1A). IDH wild-type GBM tended to have a poorer prognosis than other grade 4 gliomas (Supplementary Figure S1B). The median preoperative KPS scores were 60, 65, 70, and 80, respectively, with significantly lower KPS observed in the early surgery group (*P* < .05; Table 1 and Figure 1A, left). Simple linear regression analysis showed a positive correlation between preoperative KPS and the TTS (Figure 1A, right).

**Table 1.** Characteristics on admission of 219 GBM patients

Characteristic		TTS				
	Overall	≤ 3 days	4-7-days	8-14 days	≥ 15 days	*P*-value
Number	219	44	62	74	39	
Females	84	16	26	29	13	
Age (median)	18-91 (69)	27-91 (66)	37-90 (70)	18-88 (70)	23-82 (69)	*P * > .1*
Symptom to initial hospitalization days (95% CI)	29 (25-33)	33 (27-39)	29 (18-40)	29 (21-37)	26 (17-35)	*P * > .1**
Symptom to surgery days (95% CI)	38 (24-42)	35 (27-43)	36 (28-44)	41 (32-50)	46 (34-58)	*P * > .1**
Tumor type						
IDH wt, GBM, G4	205	41	60	71	33	*P * = .072*** (IDH wt versus others)
IDH mut, astrocytoma, G4	5	1	0	1	3	
DMG and others, G4	9	2	2	2	3	
Tumor laterality						
Right	95	21	28	34	12	*P * > .1*** (left versus others)
Left	100	17	24	34	25	
Bilateral with/without midline	23	6	10	5	2	
Midline only	1	0	0	1	0	
Tumor location						
Frontal	79	14	28	21	16	*P * > .1*** (frontal versus others)
Temporal	62	14	18	18	12	
Parietal	34	7	5	18	4	
Occipital	11	1	3	5	2	
Cerebellum	6	2	0	3	1	
Brain stem	2	1	0	1	0	
Others	25	5	8	8	4	
Median tumor maximal diameter, mm	50.8	60.5	52.1	50.3	38.8	*P *< .001*
Median tumor volume, cm3	34.0	51.9	35.1	32.3	17.1	*P *< .001*
Motor weakness						
Severe paresis	13	5	7	0	1	*P *< .001*** (no paresis versus others)
Moderate paresis	14	3	5	5	1	
Mild paresis	77	19	23	29	6	
No paresis	115	17	27	40	31	
Epilepsy						
Yes	37	3	10	8	16	*P *< .001***
No	182	41	52	66	23	
Hydrocephalus						
Yes	6	4	1	1	0	*P *= .035***
No	213	40	61	73	39	
Preoperative KPS (median)	20-100 (70)	20-90 (60)	20-100 (65)	50-100 (70)	40-100 (80)	*P * < .001*

KPS, Karnofsky Performance Status; TTS, time to surgery; ≤ 3 days, 3 days or less; ≥ 15 days, 15 days or more.

* *ANOVA Analysis;*

** *Logrank Test;*

*** *Chi-Square Test.*

Regarding the relationship between the maximal diameter of the tumor at the time of initial hospitalization and TTS, as well as the relationship between tumor volume and TTS, the values in the ≤ 3-day group were significantly larger than those in the 8-14-day and ≥ 15-day groups (Supplementary Figure S2). Only one patient developed a new lesion during the follow-up period. Regarding changes in the volume of the initial contrast-enhancing area (including the central necrotic region) and the volume just before surgery in 41 cases, the median tumor volume doubling time was 27 days, and the median growth rate was 2.5% per day (Supplementary Table S1, Supplementary Figure S3). When comparing the rate of growth between the group receiving steroids and the group not receiving them, the doubling time tended to be longer in the group receiving steroids (median value, 47.8; mean value, 940.5 ± 558.8) than non-steroid cases (median value, 26.9; mean value, 34.9 ± 18.5, *P* < .002, Student’s t test).

### Clinical Outcomes

**Figure 2.**
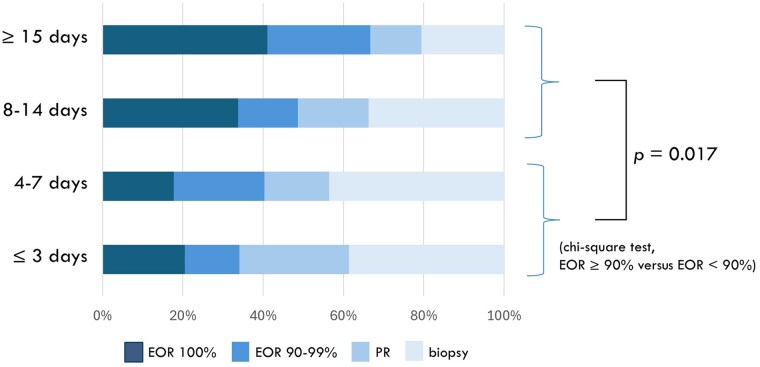
Association between time to surgery (TTS) and extent of resection (EOR). For 218 cases, excluding 2 cases with unknown resection volume, differences in EOR between two groups (≤ 3-days plus 4-7-days group versus 8-14-days plus ≥ 15-days group) were analyzed using the Mann-Whitney U test (*P* = .017).

For surgery, intraoperative photodynamic diagnosis (PDD) using 5-aminolevulinic acid was performed in 94% of cases, and intraoperative MRI was used in 43%. The shorter the TTS, the lower the utilization rates of these techniques (Supplementary Table S2). In the 8-14-day group, we identified 6 cases of anxiety or depression that required medication or counseling while patients were waiting in our hospital. In contrast, such symptoms were not observed in the ≥ 15-day group, probably because most patients in this group had been discharged home before surgery. In the analysis of EOR and related factors, EOR 100% and EOR 90-99% groups, as well as PR and biopsy groups, were combined into “EOR ≥ 90%” and “EOR < 90%”, respectively, because they were associated with similar survival outcomes (Supplementary Figure S4). In the ≤ 3-day and 4-7-day groups, 15 (34%) and 25 (40%) cases, respectively, underwent EOR ≥ 90%. These proportions were lower than those in the 8-14-day (49%) and ≥ 15-day (67%) groups (*P* = .017; Figure 2). There were association between EOR and strong luminescence on PDD and between EOR and intraoperative MRI use (Supplementary Figure S5).

Although the Kruskal-Wallis test showed no significant difference in postoperative KPS among the groups (Figure 1B, left), simple linear regression analysis revealed significantly lower postoperative KPS in cases with shorter TTS periods (Figure 1B, right). On the other hand, the difference between preoperative and postoperative KPS (ΔKPS) was significantly lower in the group with TTS ≥ 15 days compared to those with TTS ≤ 3 days and ≤ 7 days (Figure 1C, left). Simple linear regression analysis also showed that ΔKPS decreased as TTS increased, albeit with a very gentle slope (Figure 1C, right).

**Figure 3.**
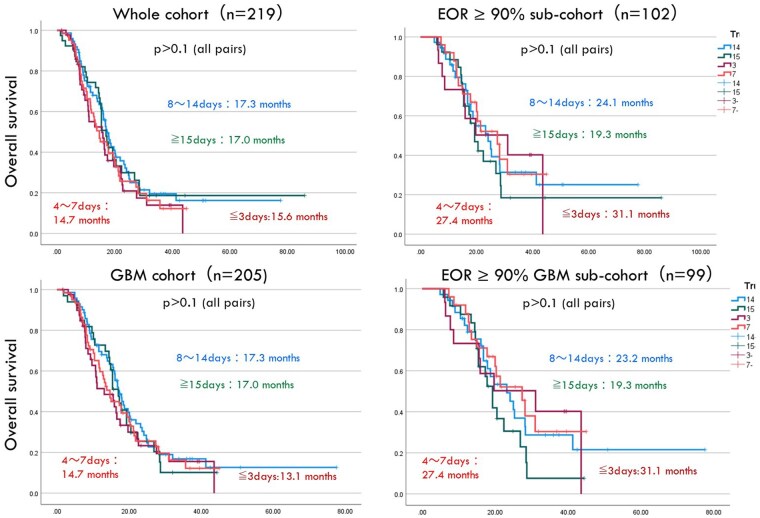
Association between time to surgery (TTS) and overall survival (OS). Upper left: entire cohort (*n* = 219); upper right: EOR ≥ 90% subcohort (*n* = 102); lower left: GBM cohort (*n* = 205); lower right: EOR ≥ 90% GBM subcohort (*n* = 99). The horizontal axis represents time in months.

Regarding postoperative treatment, combined therapy with conventional 60 Gy extended local radiotherapy (RT) and temozolomide (TMZ) was selected in 67% of cases, followed by hypofractionated RT with TMZ in 22% of cases (Supplementary Table S3). The use of conventional chemoradiotherapy tended to be lower in the early surgery groups compared with the 8-14-day and ≥ 15-day groups (*P* = .091; Supplementary Figure S6). The median OS in the entire cohort was 16.3 months (95% confidence interval (CI); 15.0-17.6 months), with no significant difference in survival among the four groups (Figure 3, upper left). Even when the analysis was limited to cases undergoing EOR ≥ 90% (Figure 3, upper right), cases with GBM, or cases with GBM undergoing EOR ≥ 90%, no significant difference was observed (Figure 3, lower).

When comparing two GBM groups based on TTS duration thresholds (one group exceeding the threshold and the other below the threshold), the groups exceeding the thresholds at 4 days, 5 days and 12 days showed statistically significantly longer survival periods than the group below the threshold (Supplementary Figure S7, Supplementary Table S4). However, no significant differences were observed at any threshold in the EOR ≥ 90% GBM sub-cohort. Furthermore, when the threshold was set at 15 days or more, almost all of groups with longer TTS had a shorter median survival time than those with the threshold was set at 14 days or less in EOR ≥ 90% GBM sub-cohort.

In univariate analysis using the Cox proportional hazards model, age, IDH status (non-GBM histology), epilepsy before the first admission, hydrocephalus on the first admission, preoperative KPS, TTS, intraoperative MRI use, MEP use, EOR ≥ 90%, postoperative KPS, ΔKPS, two-stage surgery, postoperative treatment type, and KPS at discharge yielded *P*-value < 0.1 (Table 2) and were selected as candidate factors for multivariate analysis. Two-stage surgery was correlated with hydrocephalus (*P* < .001, Fisher’s exact test). Interestingly, comparison of survival outcomes between two-stage and non-staged surgeries revealed that patients who underwent two-stage surgery had shorter survival than those who underwent non-staged surgery (Supplementary Figure S8).

**Table 2.** Univariate analyses using the Cox proportional hazards model for factors potentially affecting survival

Independent variables	Categorical variable or continuous variable	Univariate analysis			
		*P*–value	Exp(B)	95.0%, CI	
Age	continuous variable	**.012 (B = 0.016)**	**1.017**	**1.004**	**1.030**
Sex	female (versus male)	.978	–	–	–
Laterality	left (versus others)	.113	–	–	–
Lesion	frontal (versus others)	.136	–	–	–
IDH	wildtype (versus others)	**.061**	**0.504**	**0.247**	**1.031**
Tumor volume	continuous variable	.175			
Motor weakness	yes (versus no)	.105	–	–	–
Epilepsy	yes (versus no)	**.079**	**1.487**	**0.955**	**2.314**
Hydrocephalus	yes (versus no)	**.005**	**0.309**	**0.136**	**0.702**
Preoperative KPS*	continuous variable	**<.001 (B = −.017)**	**0.983**	**0.974**	**0.992**
Steroid	use (versus no use)	.406	–	–	–
TTS days	continuous variable	**.016 (B = −0.027)**	**0.974**	**0.953**	**0.995**
PDD	use (versus others)	.313	–	–	–
Intraoperative MRI	use (versus no use)	**<.001**	**1.997**	**1.445**	**2.761**
MEP	use (versus no use)	**.002**	**1.655**	**1.200**	**2.282**
Awake	use (versus no use)	.137	–	–	–
EOR	EOR ≥ 90% (versus others)	**<.001**	**2.737**	**1.977**	**3.791**
Postoperative KPS*	continuous variable	**<.001 (B = −0.032)**	**.968**	**.958**	**.978**
Delta KPS*	continuous variable	**.021 (B = −0.012)**	**0.988**	**0.978**	**0.998**
Two-staged surgery	yes (versus no)	**.036**	**0.461**	**0.224**	**0.950**
Post-operative therapy	Hypo RT+TMZ/RT only (versus conventional/others)	**<.001**	**0.406**	**0.283**	**0.581**
KPS on discharge^#^	continuous variable	**<.001 (B = −0.038)**	**0.963**	**0.954**	**0.972**

EOR, extent of resection; KPS, Karnofsky Performance Status; MEP, motor evoked potential; MRI, magnetic resonance imaging; PDD, photodynamic diagnosis; RT, radiotherapy; TMZ, temozolomide; TTS time to surgery. Bold values indicate *p* < 0.1.

Multivariate analysis demonstrated that non-GBM histology, EOR ≥ 90% and postoperative KPS were independently associated with a favorable prognosis, whereas TTS, as well as two-stage surgery, showed no significant association with OS (Table 3). Non-staged surgery and postoperative treatment using conventional chemoradiotherapy and other specialized therapies tended to result in a favorable prognosis.

**Table 3.** Multivariate analyses using the Cox proportional hazards model for factors potentially affecting survival

Independent variables	Categorical variable or continuous variable	Multivariate analysis			
		*P*-value	Exp(B)	95.0% CI	
Age	continuous variable	.626	**–**	**–**	**–**
IDH	wildtype (versus others)	**.011**	**0.343**	**0.150**	**0.781**
Epilepsy	yes (versus no)	.497	**–**	**–**	**–**
Hydrocephalus	yes (versus no)	.138	**–**	**–**	**–**
Preoperative KPS*	continuous variable	.848	**–**	**–**	**–**
TTS days	continuous variable	.532	**–**	**–**	**–**
Intraoperative MRI	use (versus no use)	.290	**–**	**–**	**–**
MEP	use (versus no use)	.389	**–**	**–**	**–**
EOR	EOR ≥ 90% (versus others)	**<.001**	**2.066**	**1.361**	**3.136**
Postoperative KPS*	continuous variable	**.011 (B = −0.019)**	**0.981**	**0.967**	**0.996**
Two-staged surgery	yes (versus no)	.099	0.504	0.223	1.138
Post-operative therapy	Hypo RT+TMZ/RT only (versus conventional/others)	.075	0.662	0.420	1.042

* Since there is a strong correlation between the ΔKPS and preoperative KPS and between the ΔKPS and postoperative KPS—the ΔKPS was excluded from multivariate analyses. And, KPS on discharge was also excluded from the independent variables in the multivariate analysis because it included cases of death. Bold values indicate *p* < 0.05.

## Discussion

### The Relationship among TTS, KPS, and EOR, and the Relationship between These Factors and Survival Outcomes

In this study, patients who underwent surgery within the early period (within 3 days) had significantly lower preoperative KPS scores. Because GBM progresses rapidly, symptoms often develop quickly, and some cases are identified during emergency department visits.^4^^,^^5^ Initial symptoms of GBM include focal neurological deficits, such as cognitive dysfunction and motor paralysis, as well as increased intracranial pressure (IICP) symptoms, including headache, nausea, and vomiting. Previous reports indicate that in newly diagnosed GBM patients aged 65 years or older, 25% were identified due to IICP-related symptoms.^8^ In particular, when IICP is difficult to control with non-surgical treatments, early surgical intervention for decompression is considered appropriate. In such cases, a low KPS score is also expected.

In this study, patients who underwent early surgery tended to include fewer EOR ≥ 90% procedures. Previous reports indicate that patients presenting through the emergency department receive an earlier diagnosis on MRI and undergo prompt tumor removal compared with those who visit the general outpatient department.^4^ This is thought to be because cases presenting through the emergency department tend to have more rapid disease progression and require earlier diagnosis and tumor debulking, particularly when symptoms such as impaired consciousness develop. In this cohort, most patients were referred to from other hospitals, and only a small number presented via the emergency department. However, among those who required surgery within 3 days, emergency admission and urgent imaging were common, suggesting a similar clinical context. In cases requiring emergency surgery, the limited time available for preoperative planning often prevented detailed consideration of the surgical approach and extent of resection. This has likely contributed to the lower resection rate. Furthermore, insufficient preoperative and intraoperative imaging, as well as inadequate intraoperative support, such as PDD, navigation systems, intraoperative MRI, and motor evoked potentials, may also contribute to a lower EOR.^2^^,^^9^

In the multivariate analysis of prognostic factors, postoperative KPS and EOR as well as non-GBM histology demonstrated significant associations with favorable survival, whereas TTS, the number of days from admission to surgery, was not identified as a prognostic factor. It is well established that both KPS and the EOR of contrast-enhancing lesions are key determinants of prognosis in GBM.^2^^,^^10^^,^^11^ It is particularly noteworthy that, despite the tendency toward lower postoperative KPS and lower EOR in cases undergoing early surgery, these factors did not significantly affect OS. Moreover, no difference in OS was observed even when the analysis was limited to EOR ≥ 90% cases. Several reports have similarly indicated that there is no difference in OS between early and non-early surgery.^6^^,^^12^ These results suggest that while emergency surgery is crucial for patients requiring early decompression, in cases where preoperative KPS is preserved and elective surgery is feasible, it is important to perform surgery aimed at achieving EOR ≥ 90% following thorough preoperative evaluation.

### When Is the Optimal Time to Perform the Surgery for WHO Grade 4 Gliomas?

In this study, although the number of days varied among cases, 82% of patients underwent surgery within 2 weeks of their initial admission date (which was nearly identical to the date of their initial MRI), and 97% underwent surgery within one month. There are no clear guidelines regarding the optimal timing of surgery for grade 4 gliomas, including GBM. High-quality evidence examining the relationship between the interval from initial imaging to surgery and patient prognosis remains limited. Most previous studies on the timing of GBM surgery have reported that approximately 75% of patients undergo surgery within three weeks,^12^ and 82-86% undergo surgery within 1 month.^6^^,^^13^ Analysis of the group with a preoperative interval of 15 days or longer revealed a significantly higher proportion of grade 4 gliomas other than typical IDH-wildtype GBM. Cases of atypical grade 4 glioma have been reported to exhibit non-typical imaging characteristics.^14^^,^^15^ A report examining imaging differences between IDH-wildtype GBM and IDH-mutant astrocytoma indicates that the latter exhibits more frequent intratumoral hemorrhage, involves cortical lesions, and has lower ADC values.^15^ Such atypical imaging findings may have contributed to the prolonged preoperative interval.

In this study, the median tumor volume doubling time and the median growth rate were 27 days and 2.5% per day, although there was substantial variability among patients. Previous studies have also examined tumor growth rates. In untreated GBM, tumor volume, including both the contrast-enhancing region and the central necrotic area, has been reported to double within a median of 21-48 days,^7^^,^^16^^,^^17^ showing no marked difference from the findings of this study. With respect to growth rates, previously reported median values were 1.4% and 2.1%, whereas our study demonstrated slightly higher rates. Müller and colleagues have suggested that surgery performed within one month does not influence prognosis, regardless of tumor volume or growth rate.^6^

However, for tumors exceeding 50 mL in volume, the resection rate tends to be lower and survival time shorter.^6^ Another paper shows that in comparison between tumor volume in the early diagnosis group (radiologically diagnosed within 14 days from the initial symptoms) and late diagnosis group (15 days or later from symptom), the median tumor volumes of the late diagnosis group (29.0 cm^3^) was larger than that of the early diagnosis group (12.0 chm^3^).^18^ Furthermore, our analysis revealed that when TTS exceeded 2 weeks, mOS showed a slight decrease even in the EOR ≥ 90% GBM subcohort. Therefore, an interval of approximately 2 weeks before surgery is likely to be optimal. However, it should be noted that EOR ≥ 90% and postoperative KPS are the strongest prognostic factors, and TTS itself does not directly influence prognosis.

In this study, tumor volume doubling time on preoperative MRI tended to be longer in the steroid group than in the non-steroid group. Previous reports have suggested that steroid use may influence tumor growth.^19^ Therefore, in patients who require a delay before surgery, steroid use may be considered; however, caution is warranted in cases where lesions are difficult to distinguish from malignant lymphoma or in patients with severe diabetes. In our study, although two-stage surgery was associated with worse prognosis in the univariate analysis, multivariate analysis suggested that, similar to TTS, it was not independently associated with survival. Two-stage surgery was correlated with hydrocephalus, and it is likely that tumors complicated by hydrocephalus influence survival through ­multiple factors, including tumor location and extent of resection. Therefore, in patients with hydrocephalus requiring urgent management, an approach in which hydrocephalus is first treated, with or without biopsy, followed by tumor resection once the patient’s condition stabilizes may be considered.

### Limitations of This Study

In terms of limitations, this study was retrospective, and the timing of surgery was determined based on a comprehensive assessment of clinical symptoms and imaging findings. Furthermore, TTS was defined as the period from the first admission to our hospital to the date of surgery. As most patients were referred to from other institutions, TTS is likely underestimated compared with the actual interval from the original initial presentation to surgery. For prognostic evaluation, we used KPS and OS. However, cognitive function and the degree of higher brain dysfunction also substantially affect a patient’s quality of life. Information on these factors was insufficient, making it difficult to incorporate them into the analysis. Although the length of hospital stays and hospitalization costs are clinically important factors, they were not included in this analysis because they are influenced by variables such as the duration of radiation therapy and the time required to arrange transfer to another facility. When calculating doubling time, we measured the contrast-enhancing area, including the central necrotic region, on contrast-enhanced T1-weighted images. In some cases, however, different MRI machines were used between the initial examination and the preoperative period, which may have led to inaccuracies in measurement. In addition, this study did not assess the extent of tumor infiltration represented by high-signal areas on peripheral T2-weighted images. The timing of postoperative chemoradiotherapy were also not examined, although some reports suggest that delayed postoperative chemotherapy is associated with poor prognosis.^20^ This study analyzed the use of PDD, intraoperative MRI, and electrophysiological monitoring as intraoperative support; however, it did not examine factors such as the presence or absence of preoperative and intraoperative nerve fiber mapping, which represents an additional limitation.

## Conclusions

In the surgical management of grade 4 gliomas, including GBM, thorough preoperative evaluation is essential because the extent of resection is closely associated with survival outcomes. In addition, careful attention must be given to preserving neurological function in order to maintain postoperative KPS. To achieve these goals effectively, it is ideal to plan surgery at an appropriate time that considers KPS preservation and maximal safe resection, except in cases where emergency intervention is unavoidable.

## Supplementary Material

vdag133_Supplementary_Data

## Data Availability

Raw data were generated at the Department of Neurosurgery, University of Tsukuba. Derived data supporting the results of this study are available from the corresponding author (E.I.) on request.
