# Supplementary material for: Association between time to surgery and survival in patients with initially diagnosed WHO 2021 grade 4 gliomas
Source: Neurooncol Adv. 2026 May 20;8(1):vdag133. doi: 10.1093/noajnl/vdag133 (PMC13264394; doi:10.1093/noajnl/vdag133)

Association between Time to Surgery and Survival in Patients with Initially Diagnosed WHO 2021 Grade 4 Gliomas

|  | **Median values** | **Mean value ±SE** |
| --- | --- | --- |
| **Doubling time*, days** | 27.0 | 211.6 ± 118.8 |
| **Growth rate*, mm^3^ / day** | 397.1 | 485.9 ± 97.6 |
| **Growth rate*, % / day** | 2.45 | 2.47 ± 0.35 |

**Suppl. Table 1. Preoperative GBM growth rates.**

* *Forty-one cases in which an MRI scan was performed at least 10 days after the initial head MRI and prior to surgical resection.

**Suppl. Table2. Surgical devices used and extent of resection in 219 GBM patients.**

| Characteristic | |  | TTS | | | |  |
| --- | --- | --- | --- | --- | --- | --- | --- |
|  |  | Overall | ≤ 3 days | 4-7 days | 8-14 days | ≥ 15 days | *p* value |
| Number | | 219 | 44 | 62 | 74 | 39 |  |
| Steroid use | | | | | | | |
|  | Used | 34 (16%) | 6 (14%) | 9 (15%) | 16 (22%) | 3 (8%) | *p>*0.1# (used versus not used) |
|  | Not used | 185 (84%) | 38 (86%) | 53 (85%) | 58 (78%) | 36 (92%) |  |
| Intraoperative PDD | | | | | | | |
|  | Used | 206 (94%) | 37 (84%) | 59 (95%) | 71 (96%) | 39 (100%) | *p*=0.012# (used versus others) |
|  | Strong | 154 | 28 | 42 | 56 | 28 |  |
|  | Vague/positive | 35 | 5 | 11 | 12 | 7 |  |
|  | Negative | 17 | 4 | 6 | 3 | 4 |  |
|  | Not used/ unknown | 13 (6%) | 7 (16%) | 3 (5%) | 3 (4%) | 0 (0%) |  |
| Intraoperative MRI | | | | | | | |
|  | Used | 94 (43%) | 6 (14%) | 18 (29%) | 44 (59%) | 26 (67%) | *p*<0.001# (used versus not used) |
|  | Not used | 125 (57%) | 38 (84%) | 44 (71%) | 30 (41%) | 13 (33%) |  |
| Intraoperative neurophysiological monitoring / Awake surgery | | | | | | | |
|  | Used | 90 (41%) | 12 (27%) | 25 (40%) | 33 (45%) | 20 (51%) | *p>*0.1# (used versus not used) |
|  | MEP with/without SEP | 89 | 12 | 25 | 33 | 19 |  |
|  | Awake surgery | 3## | 0 | 0 | 0 | 3## |  |
|  | Not used | 129 (59%) | 32 (73%) | 37 (60%) | 41 (55%) | 19 (49%) |  |
| Steroid use | | | | | | | |
|  | Used | 34 (15%) | 6 (13%) | 9 (15%) | 16 (22%) | 3 (8%) | *p>*0.1# (used versus not used) |
|  | Not used | 185 (84%) | 38 (86%) | 53 (85%) | 58 (78%) | 36 (92%) |  |
| Anxiety/ depression | | | | | | | |
|  | Yes | 6 | 0 | 0 | 6 | 0 | *p*<0.007# (yes versus no) |
|  | No | 213 | 44 | 62 | 68 | 39 |  |
| EOR | | | | | | | |
|  | EOR ≥ 90 | 61 (28%) | 9 (20%) | 11 (18%) | 25 (34%) | 16 (41%) | *p*=0.017# (EOR ≥ 90 versus < 90, see Figure3) |
|  |  | 41 (19%) | 6 (14%) | 14 (23%) | 11 (15%) | 10 (26%) |  |
|  | EOR < 90 | 40 (18%) | 12 (27%) | 10 (16%) | 13 (18%) | 5 (13%) |  |
|  |  | 77 (35%) | 17 (39%) | 27 (44%) | 25 (34%) | 8 (21%) |  |

EOR, extent of resection; KPS, Karnofsky Performance Status; MEP, motor evoked potential; MRI, magnetic resonance imaging; OS, overall survival; PDD, photodynamic diagnosis; PR, partial resection; SEP, somatosensory evoked potential; TMZ, temozolomide; TTS time to surgery, #chi-square test, ## Two cases are combined with MEP monitoring

**Suppl. Table3. Outcomes in 219 GBM patients.**

| Characteristic | |  | TTS | | | |  |
| --- | --- | --- | --- | --- | --- | --- | --- |
|  |  | Overall | ≤ 3 days | 4-7 days | 8-14 days | ≥ 15 days | *p* value |
| Number | | 219 | 44 | 62 | 74 | 39 |  |
| Postoperative KPS (median) | | 20-100 (70) | 20-90  (60) | 40-100 (60) | 30-100  (70) | 30-90  (70) | *p*>0.1* |
| Complications | | | | | | | |
|  | Extracranial complications* (%) | 9 (4%) | 2 (5%) | 1 (2%) | 4 (5%) | 2 (5%) | *p*>0.1** |
|  | Epilepsy/acute symptomatic seizure (%) | 8 (4%) | 2 (5%) | 2 (3%) | 2 (3%) | 2 (5%) | *p*>0.1** |
|  | Other intracranial transient/mild/moderate symptoms or complications** (%) | 57 (26%) | 8 (18%) | 18 (29%) | 18 (24%) | 13 (33%) | *p*>0.1** |
|  | Mortality (excluding tumor progression) | 0 | 0 | 0 | 0 | 0 | *p*>0.1** |
| Two-stage surgery | | | | | | | |
|  | Yes | 12 | 8 | 2 | 1 | 1 | *P<*0.001** (yes versus no) |
|  | No | 207 | 36 | 60 | 73 | 38 |  |
| Postoperative treatments | | | | | | | |
|  | Conventional chemoradiotherapy (TMZ+ 60Gy RT) | 145 (66%) | 26 (59%) | 36 (58%) | 52 (70%) | 31 (79%) | *P=*0.091** (conventional versus others, see Figure5) |
|  | Hypo-fractionated RT + TMZ | 48 (22%) | 12 (27%) | 20 (32%) | 11 (15%) | 5 (13%) |  |
|  | RT only | 3 (1%) | 1 (2%) | 0 (0%) | 1 (1%) | 1 (3%) |  |
|  | Other special therapies including proton therapy and immunotherapy, etc. | 23 (11%) | 5 (11%) | 6 (10%) | 10 (14%) | 2 (5%) |  |
| KPS at discharge (median) | | 0-100 (70) | 30-90 (70) | 30-90 (60) | 30-100 (70) | 0-100 (70) | *p*>0.1* |
| Place of discharge | | | | | | | |
|  | Home | 143 (65%) | 28 (64%) | 35 (56%) | 54 (73%) | 26 (67%) | *p*>0.1** (home versus others) |
|  | Elderly Care Facilities/ Long-Term Care Beds | 53 (24%) | 11 (24%) | 22 (35%) | 12 (16%) | 8 (21%) |  |
|  | Recovery-Phase Rehabilitation Hospital | 7 (3%) | 1 (2%) | 3 (5%) | 2 (3%) | 1 (3%) |  |
|  | Others/unknown | 14 (6%) | 4 (9%) | 2 (3%) | 6 (8%) | 2 (5%) |  |
|  | Dead during hospitalization | 2 (1%) | 0 (0%) | 0 (0%) | 0 (0%) | 2 (5%) |  |

KPS, Karnofsky Performance Status; MRI, magnetic resonance imaging; RT, radiotherapy; TMZ, temozolomide; TTS time to surgery, * ANOVA analysis; ** chi-square test

**Suppl. Table4. Differences in overall survival time in GBM group and EOR ≥ 90 GBM group when various TTS durations are used as thresholds.**

| **days of TTS** | **GBM group** | | | **EOR ≥ 90 GBM group (n=99)** | | |  |
| --- | --- | --- | --- | --- | --- | --- | --- |
|  | **median OS of Lower TTS group** | **median OS of Higher TTS group** | **P value** | **median OS of Lower TTS group** | **median OS of Higher TTS group** | **P value** | |
| **≦3 versus >3** | **13.1** | **16.8** | **p>0.1** | **31.1** | **20.5** | **p>0.1** | |
| **≦4 versus >4** | **11.0** | **17.3** | **p=0.020** | **31.1** | **20.5** | **p>0.1** | |
| **≦5 versus >5** | **12.7** | **17.3** | **p=0.010** | **20.3** | **21.4** | **p>0.1** | |
| **≦6 versus >6** | **13.5** | **17.0** | **p>0.1** | **28.2** | **20.5** | **p>0.1** | |
| **≦7 versus >7** | **13.8** | **17.0** | **p>0.1** | **27.4** | **19.7** | **p>0.1** | |
| **≦8 versus >8** | **14.6** | **17.8** | **p>0.1** | **21.4** | **19.7** | **p>0.1** | |
| **≦9 versus >9** | **14.7** | **17.3** | **p>0.1** | **21.4** | **19.7** | **p>0.1** | |
| **≦10 versus >10** | **15.0** | **17.3** | **p>0.1** | **23.2** | **19.7** | **p>0.1** | |
| **≦11 versus >11** | **15.5** | **17.3** | **p>0.1** | **23.2** | **19.3** | **p>0.1** | |
| **≦12 versus >12** | **15.5** | **17.8** | **p=0.023** | **21.4** | **20.5** | **p>0.1** | |
| **≦13 versus >13** | **15.6** | **17.3** | **p>0.1** | **23.2** | **19.3** | **p>0.1** | |
| **≦14 versus >14** | **16.1** | **17.0** | **p>0.1** | **24.1** | **19.3** | **p>0.1** | |
| **≦15 versus >15** | **16.0** | **17.0** | **p>0.1** | **23.2** | **19.3** | **p>0.1** | |
| **≦16 versus >16** | **15.9** | **19.3** | **p>0.1** | **22.4** | **19.3** | **p>0.1** | |
| **≦17 versus >17** | **15.7** | **19.3** | **p>0.1** | **22.4** | **19.3** | **p>0.1** | |
| **≦18versus >18** | **16.0** | **17.8** | **p>0.1** | **22.4** | **17.8** | **p>0.1** | |
| **≦19 versus >19** | **16.0** | **17.8** | **p>0.1** | **22.4** | **17.8** | **p>0.1** | |
| **≦20 versus >20** | **16.0** | **17.0** | **p>0.1** | **21.4** | **19.3** | **p>0.1** | |
| **≦21 versus >21** | **16.1** | **17.0** | **p>0.1** | **21.4** | **15.4** | **p>0.1** | |
| **≦22 versus >22** | **16.1** | **17.0** | **p>0.1** | **21.4** | **15.4** | **p>0.1** | |
| **≦23 versus >23** | **16.1** | **17.0** | **p>0.1** | **21.4** | **15.4** | **p>0.1** | |
| **≦24 versus >24** | **16.1** | **17.0** | **p>0.1** | **21.4** | **15.4** | **p>0.1** | |
| **≦25 versus >25** | **16.1** | **15.4** | **p>0.1** | **21.4** | **15.4** | **p>0.1** | |

**Suppl. Figure 1. Association between time to surgery (TTS) and tumor types.**

A. Fisher's exact test was performed to compare the proportion of IDH-wildtype cases between the group with 15 days or more (≥ 15-days) and the group with less than 15 days (*p* = 0.022). B. Survival curves for patients with IDH wild-type GBM and other grade 4 gliomas are shown.


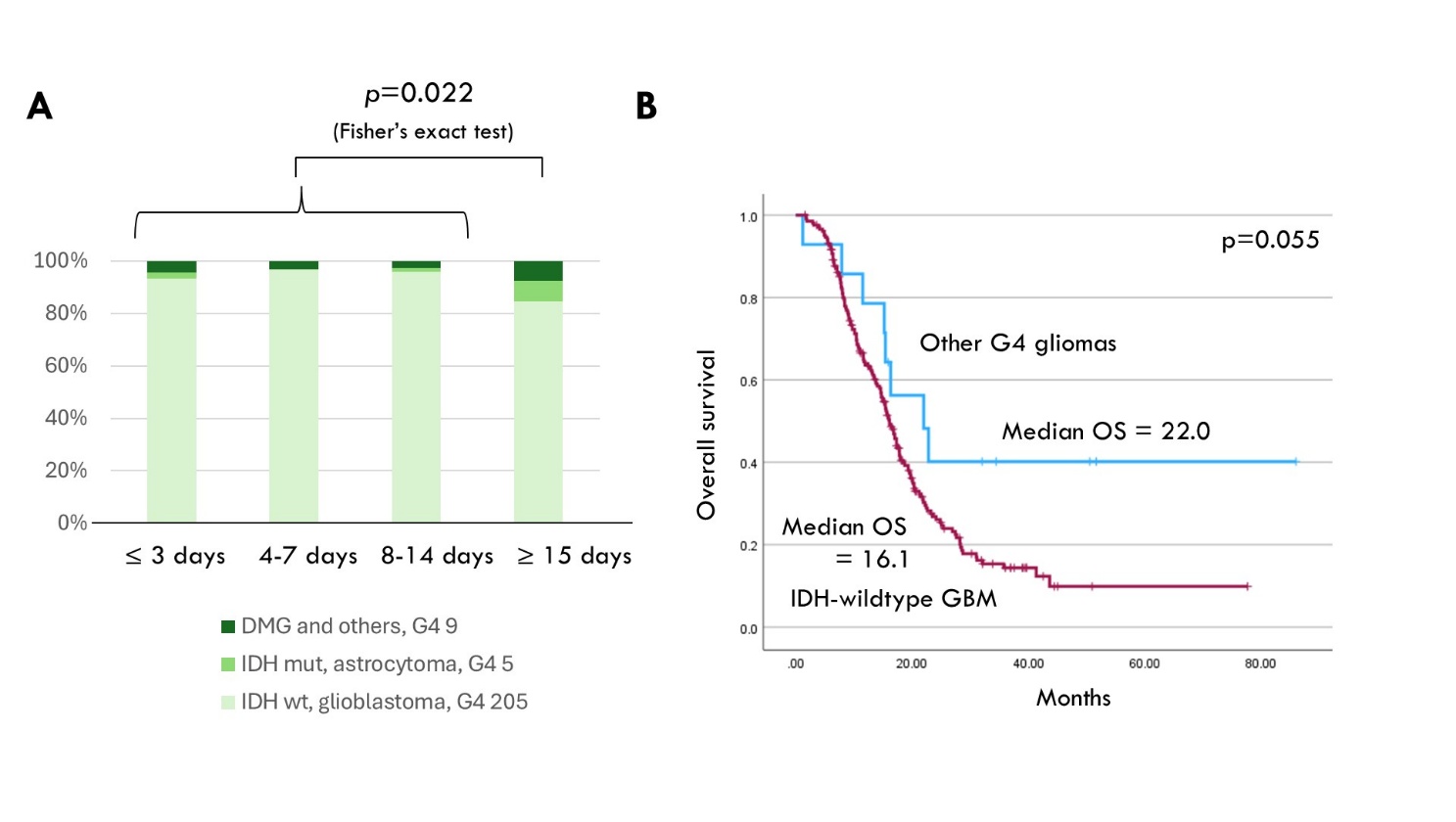


**Suppl. Figure 2. Relationship between the maximal diameter of the tumor at the time of initial hospitalization and TTS (left), as well as the relationship between tumor volume and TTS (right).**

**The Kruskal-Wallis test with Bonferroni correction was performed for the left and right panels, respectively.**


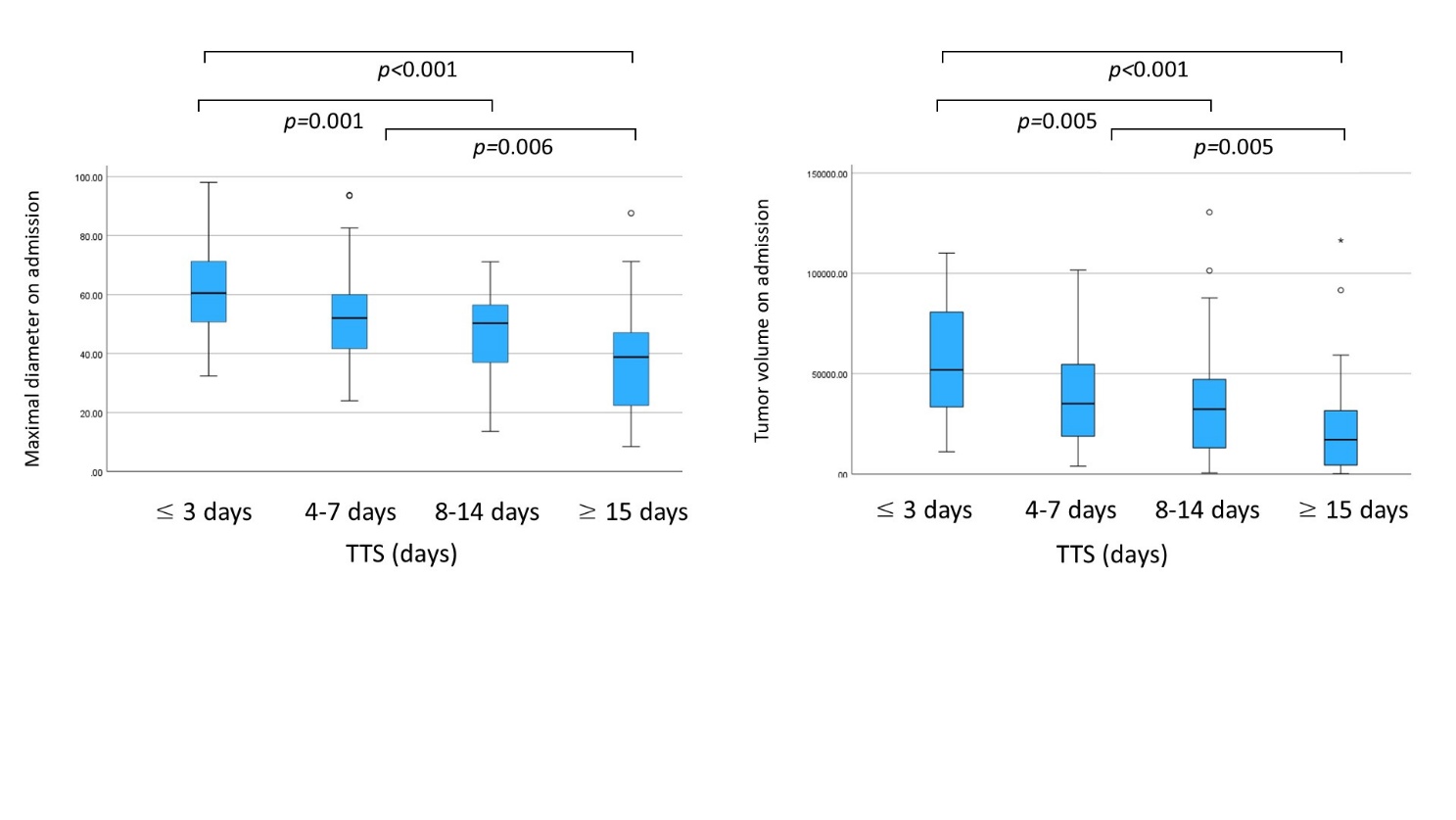


**Suppl. Figure 3. The relationship between MRI scan interval and the natural logarithm (ln) of the ratio of follow-up MRI volume to initial MRI volume**


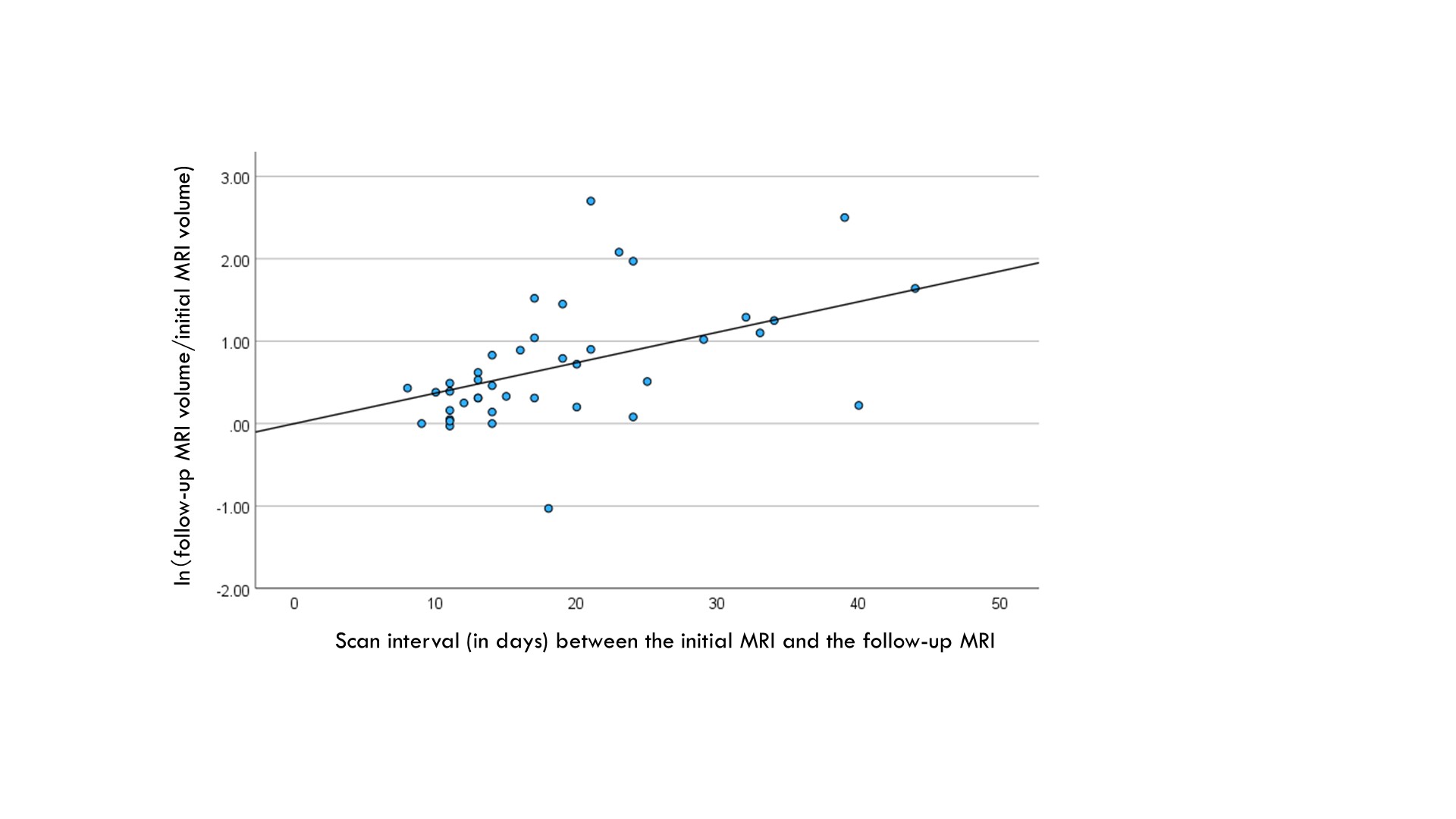


**Suppl. Figure 4. The relationship between extent of resection and overall survival**


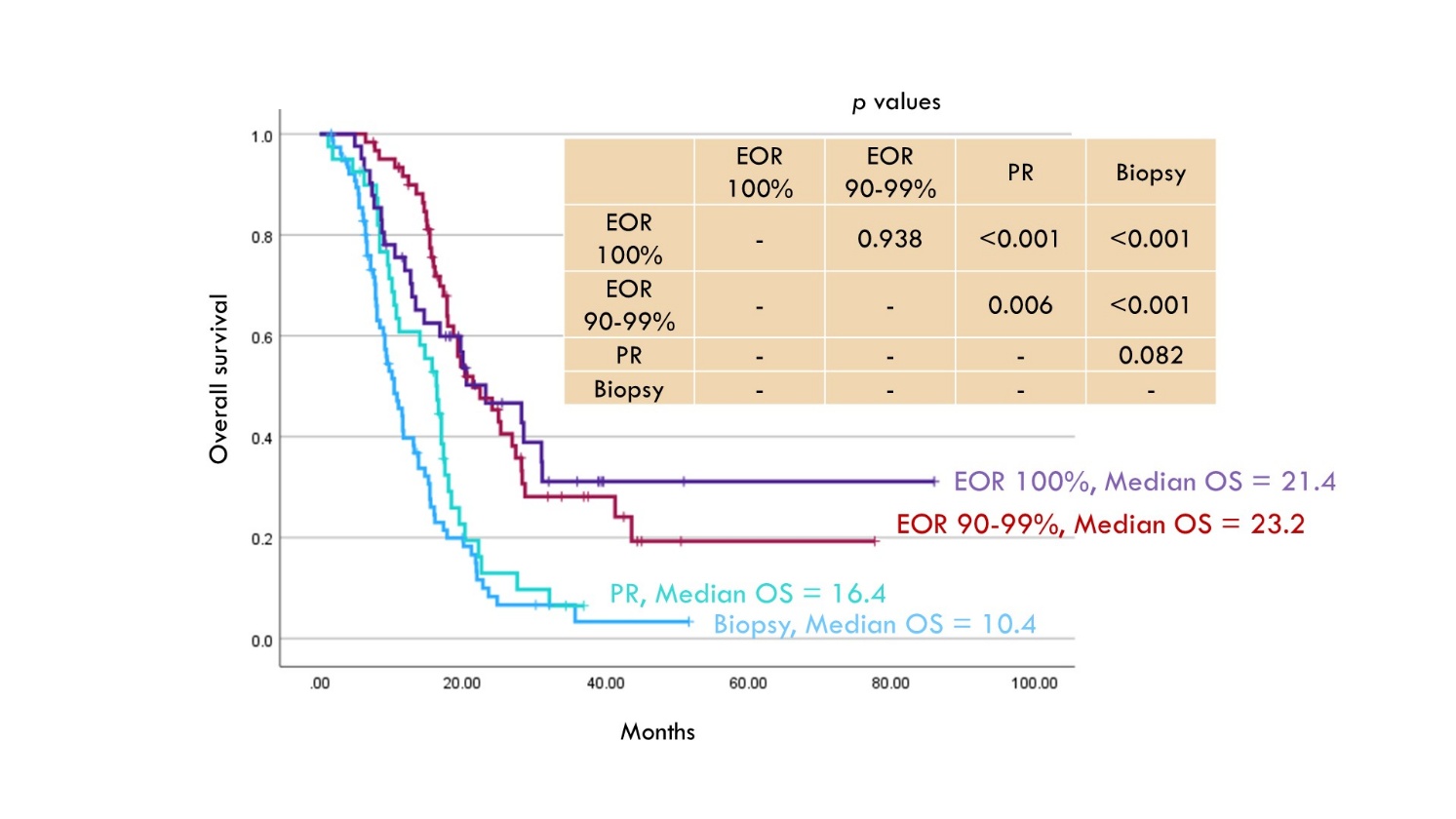


**Suppl. Figure 5. Association between intraoperative support and extent of resection (EOR).**For 219 cases differences in EOR (STR/GTR vs. PR/biopsy) were analyzed according to the use of PDD (upper panel) and intraoperative MRI (lower panel) using the chi-square test.


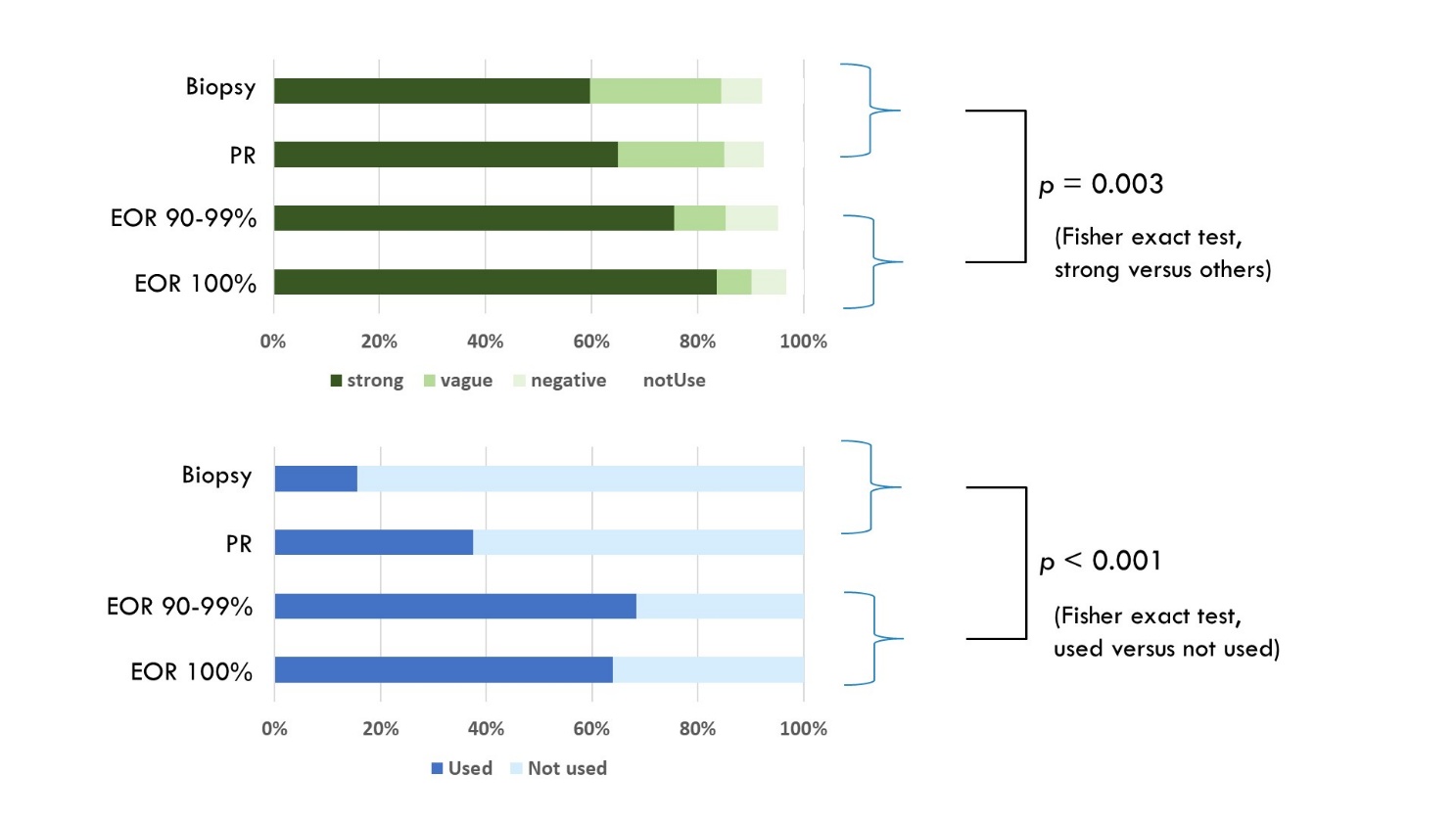


**Suppl. Figure 6. Association between time to surgery (TTS) and the type of postoperative treatment.**

For 220 cases, differences in postoperative treatment between two groups (≤ 3-days plus 4–7-days group versus 8–14-days plus ≥ 15-days group) were analyzed using the chi-square test.
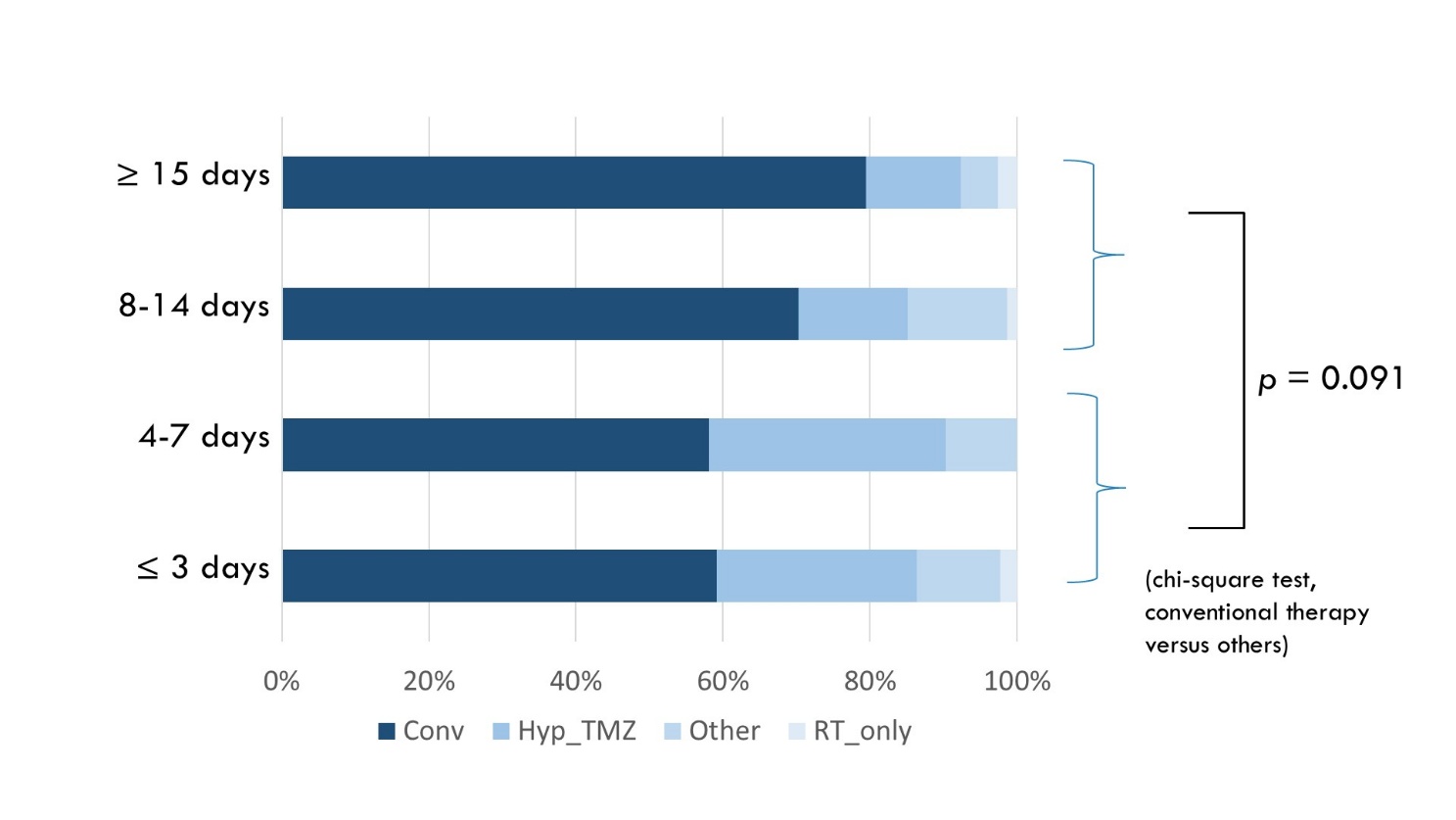


**Suppl. Figure 7. Differences in overall survival time when various TTS durations are used as thresholds.**

A and B. Median overall survival time in the two-group comparison when each duration from 3 to 25 days is used as a threshold. (A, upper: GBM cohort; B, lower: EOR ≥ 90 GBM subgroup.) In the upper figures, * indicates thresholds showing significant differences (p<0.05) by log-rank test. In the lower figure, no significant differences were observed in any of the two-group comparisons. C and D. Survival curves for the GBM cohort and the EOR ≥ 90 GBM subcohort at representative thresholds. (left: TTS 5 days as threshold; right: TTS 12 days as threshold)


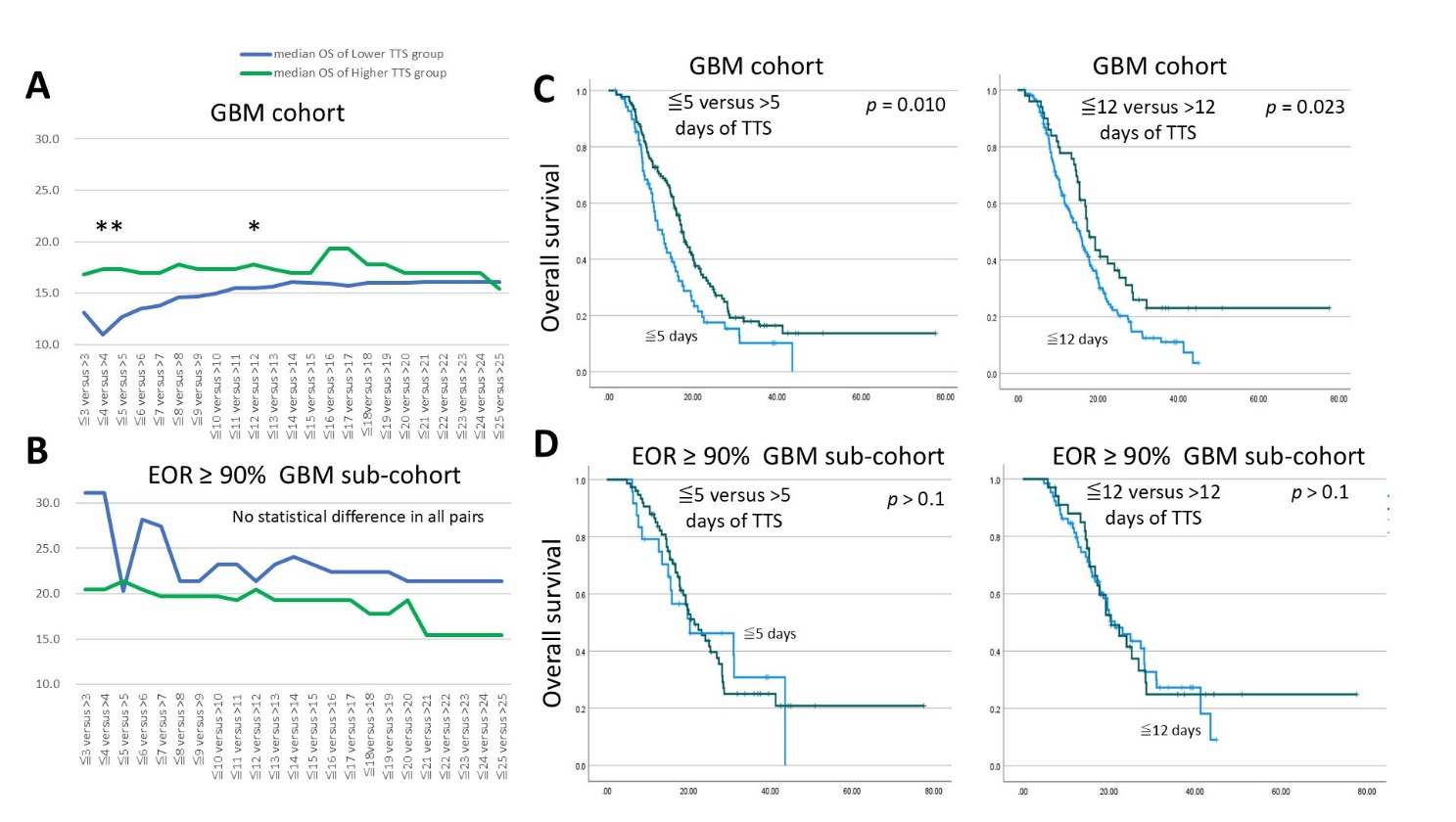


**Suppl. Figure 8. A comparison of survival outcomes between two-staged and non-staged surgery**
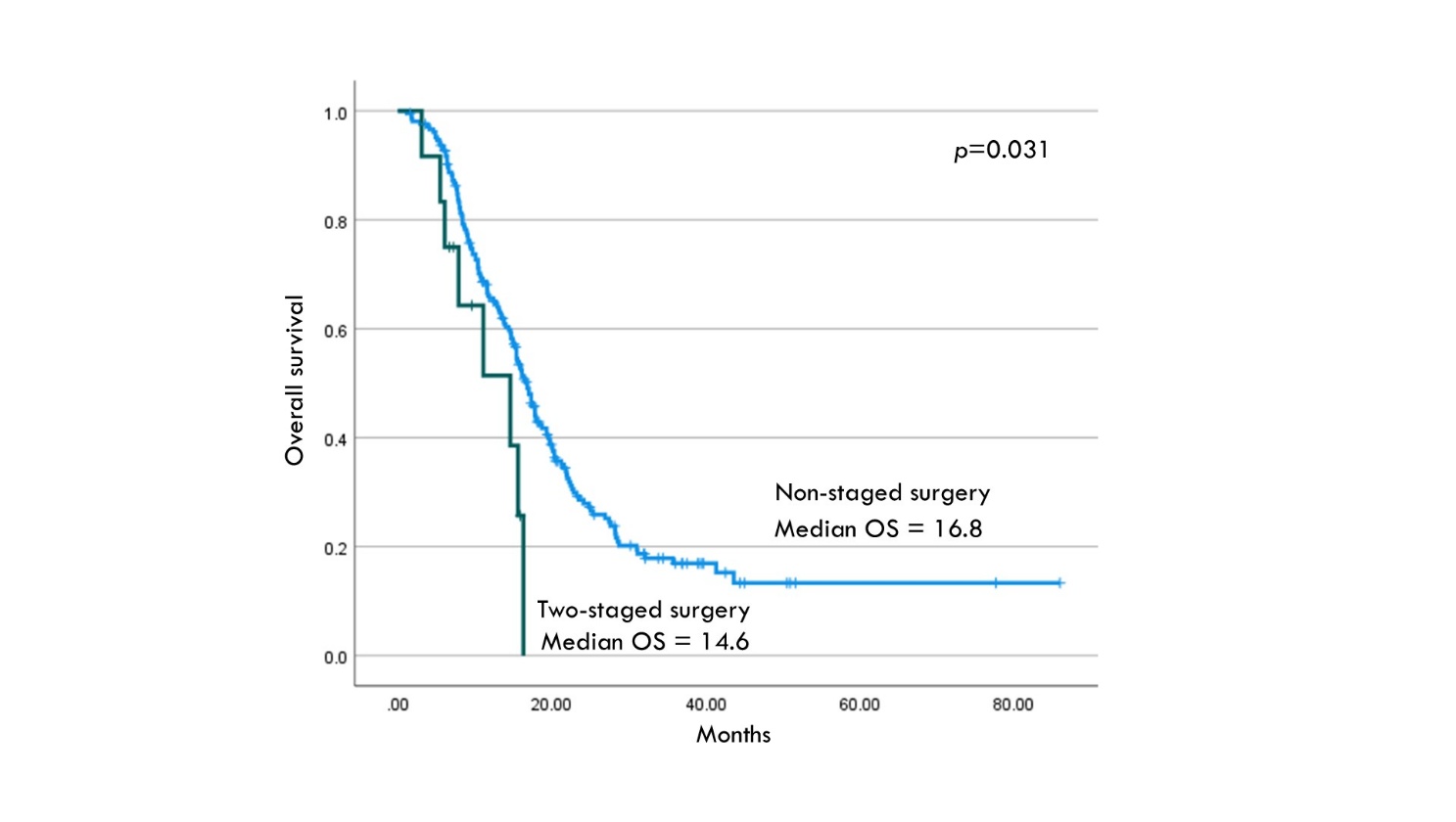

Supplement: vdag133_Supplementary_Data [file vdag133_supplementary_data.docx]
